# Positioning for success: building capacity in academic competencies for early-career researchers in sub-Saharan Africa

**DOI:** 10.1017/gmh.2019.14

**Published:** 2019-07-19

**Authors:** C. Merritt, H. Jack, W. Mangezi, D. Chibanda, M. Abas

**Affiliations:** 1 King's College London, Institute of Psychiatry, Psychology and Neuroscience, London, UK; 2 University of Washington, School of Medicine, Seattle, USA; 3 Department of Psychiatry, University of Zimbabwe College of Health Sciences, Harare, Zimbabwe

**Keywords:** Africa, capacity building, career development, research, training

## Abstract

**Background.:**

Capacity building is essential in low- and middle-income countries (LMICs) to address the gap in skills to conduct and implement research. Capacity building must not only include scientific and technical knowledge, but also broader competencies, such as writing, disseminating research and achieving work–life balance. These skills are thought to promote long-term career success for researchers in high-income countries (HICs) but the availability of such training is limited in LMICs.

**Methods.:**

This paper presents the contextualisation and implementation of the Academic Competencies Series (ACES). ACES is an early-career researcher development programme adapted from a UK university. Through consultation between HIC and LMIC partners, an innovative series of 10 workshops was designed covering themes of self-development, engagement and writing skills. ACES formed part of the African Mental Health Research Initiative (AMARI), a multi-national LMIC-led consortium to recruit, train, support and network early-career mental health researchers from four sub-Saharan African countries.

**Results.:**

Of the 10 ACES modules, three were HIC-LMIC co-led, four led by HIC facilitators with LMIC training experience and three led by external consultants from HICs. Six workshops were delivered face to face and four by webinar. Course attendance was over 90% and the delivery cost was approximately US$4500 per researcher trained. Challenges of adaptation, attendance and technical issues are described for the first round of workshops.

**Conclusions.:**

This paper indicates that a skills development series for early-career researchers can be contextualised and implemented in LMIC settings, and is feasible for co-delivery with local partners at relatively low cost.

## Background

### Research capacity building in low- and middle-income countries

Relative to their large populations and a high burden of disease, low- and middle-income countries (LMICs) experience a significant healthcare treatment gap, in part due to weak health systems (Chu *et al*. 2014). This is particularly the case with mental health in LMICs, where it is estimated that 90% of people do not receive treatment (Thornicroft *et al*. 2012). One way to help strengthen health systems is through improving health research capacity, which facilitates the development of a local evidence base for health interventions, a stronger healthcare workforce, and the capacity to rigorously evaluate interventions (Saraceno & Saxena, 2004; Liu *et al*. 2016; Schneider *et al*. 2016). Funding for health research is disproportionately low in LMICs (Thornicroft *et al*. 2012; Kebede *et al*. 2014; World Bank, 2014), which often means that there is little support for research training or incentive to enter research careers. Additionally, LMICs are under-represented in research publications. For example, it is estimated that <1% of biomedical research papers originate in Africa, despite the continent being home to nearly one-fifth of the world's population (Chu *et al*. 2014). Accordingly, there is also a great need to strengthen local research capacity and support early-career researchers from LMICs to be competitive for international research grants (Cochrane *et al*. 2014). Research training enables LMIC researchers to produce high-quality research, impact their local and national health systems, develop sustainable careers to mitigate ‘brain drain’ out-migration of talented researchers and help build strong healthcare and research institutions (Hagopian *et al*. 2004; Whitworth *et al*. 2008; Thornicroft *et al*. 2012; Kebede *et al*. 2014; World Bank, 2014; Piette *et al*. 2015; Sheehan *et al*. 2015; Uthman *et al*. 2015; Marjanovic *et al*. 2017). However, there is a lack of data on research capacity building in LMICs (Thornicroft *et al*. 2012; Cochrane *et al*. 2014; Liu *et al*. 2016).

Several major programmes in LMICs have recently targeted research capacity building and strengthening, such as the South Asian Hub for Advocacy, Research and Education on Mental Health (SHARE; Sharma & Razzaque, 2017) and the Latin American Treatment and Innovation Network in Mental Health (LATIN-MH; Bonini *et al*. 2017). Key examples in sub-Saharan Africa (SSA) are the Medical Education Partnership Initiative [MEPI; including Improving Mental Health Education and Research capacity in Zimbabwe (IMHERZ)], Emerging Mental Health Systems in Low- and Middle-Income Countries (EMERALD) and two National Institute of Mental Health (NIMH) multi-centre collaborative projects: Africa Focus on Intervention Research for Mental Health (AFFIRM) and the Partnership for Mental Health Development in Sub-Saharan Africa (PaM-D) (Abas *et al*. 2014; Piette *et al*. 2015; Pilowsky *et al*. 2016; Schneider *et al*. 2016; Hakim *et al*. 2018; Hanlon *et al*. 2018; Semrau *et al*. 2018). These programmes addressed research capacity building both at the system and individual levels with technical training (e.g. methods, statistics, evaluation, care models), and included some modules for non-technical career development skills, such as mentoring, teaching and grant-writing. However, none offered their participants a broad package that encompassed a range of self-development, engagement and writing skills.

### Career development for individual researchers

At the individual researcher level, these non-technical abilities are needed to build a sustainable career (Alpay & Walsh, 2008). The WHO's ESSENCE framework for research capacity building delineates capacity in three areas: (1) doing research (technical knowledge, methods); (2) managing research (funding, plans and reports); and (3) applying and sharing research results (dissemination and implementation activities such as publication, policy engagement, multi-stakeholder consultancy) (World Health Organisation, 2016). ESSENCE reflects the need for broader competencies in a modern research career, beyond technical scientific skills. This is echoed in capacity building literature which emphasises the importance of researcher self-development (mentoring, work–life balance, career strategy and team working skills), writing skills (both for academic publication and grant applications) and engagement (policy influence, media dissemination, teaching and presentations) (Bland & Schmitz, 1986; Lansang & Dennis, 2004; Debowski, 2012; Vitae, 2012; Ionescu *et al*. 2017; Semrau *et al*. 2018). Together, all of these abilities should allow researchers to position themselves for success over a long-term career. However, there are limited empirical data from either high-income countries (HICs) or LMICs on schemes which have included these broader elements.

Mentoring is perhaps the career development skill which has received the most attention in the literature on researcher development. A meta-analysis found that being mentored in academia was associated with greater publication output and grant success (Sambunjak *et al*. 2006), while two qualitative studies noted its general perceived impact on researcher wellbeing and career sustainability (Angelique *et al*. 2002; Iversen *et al*. 2014). One UK evaluation among female academics found that mentoring significantly increased job-related wellbeing, self-esteem and self-efficacy (Dutta *et al*. 2011). There are indications that demand for mentoring in SSA is high. A recent pilot survey in Ghana found that nearly half of female health scientists wanted to receive mentoring; the top issue of concern was combining a science career with a family (Obiri-Yeboah *et al*. 2016).

Other literature offers opinions on finding and maintaining good work–life balance, the importance of teamwork and communication, and writing skills both for publications and grants (Debowski, 2012; Ionescu *et al*. 2017). However, the majority of publications on the implementation of training in these broader skills for research careers come from HICs, and most rely on anecdotal evidence of impact (Glass *et al*. 2014). Moreover, many programmes provide only one or two of these career development skills as an adjunct to technical research training, rather than a full package of career development skills.

Notable instances can be found in SSA where career development skills have been incorporated into health research capacity building programmes. Perhaps the most comprehensive of these was the Afya Bora Consortium package which, alongside technical skills, trained African health leaders in communication, leadership and grant writing. Qualitative evaluation of the 16 Ugandan medical fellows trained indicated perceived benefits in terms of innovations in practice and quality improvements, with anecdotal reports of grant success (Nakanjako *et al*. 2015). The Economic Community of West African States developed the Young Professionals Internship Programme (YPIP), based in Burkina Faso, to train regional health workers in leadership, teamwork, negotiation, strategic planning and advocacy. While no evaluation of these courses is presented, the programme has a high post-completion employment rate, with nearly half of its graduates becoming healthcare programme coordinators (Sanou *et al*. 2014). The IMHERZ project in Zimbabwe incorporated mentoring into a broader medical education training programme (Hakim *et al*. 2018). While these examples are encouraging, they are few and far between, and like their HIC counterparts, do not combine a broad range of career development skills into a single package.

Accordingly, we use the example of the African Mental Health Research Initiative (AMARI) in this paper to describe the development and implementation of an innovative capacity-building course that focuses on broader, non-technical research competencies. We present the course components in detail and assess the challenges and opportunities in adapting a course from a high-income setting for collaborative delivery in SSA with local partners.

## Method

### Context

One of the largest LMIC research capacity building initiatives is the Wellcome Trust's DELTAS programme (Developing Excellence in Leadership, Training and Science), of which one programme is AMARI. AMARI aims to recruit and train a network of 50 early-career researchers at M.Phil., Ph.D. and Post-doctoral levels across four countries: Ethiopia, Malawi, South Africa and Zimbabwe. Research fellows are recruited from a range of health science and policy work fields (e.g. nursing, medicine, psychiatry, occupational therapy, clinical psychology, physiotherapy) and most will graduate from departments of health science, public health or psychiatry within their research institutions. Details of the recruitment process are given in online Supplementary Appendix 1. AMARI also aims to support the creation of sustainable career paths for these young scientists to ensure maximum long-term benefit to their countries' health sectors.

In addition to a broad package of support, which includes research methods training, AMARI fellows are given training to assist career development. These transferable skills (including domains of self-development, engagement and writing) are advocated for success and impact both nationally, on health systems and policy, and internationally in securing competitive research funding (Glass *et al*. 2014). Training workshops were modified from early-career researcher development courses in HICs for collaborative delivery between HIC and LMIC partners in AMARI, as recommended in capacity building literature (Curry *et al*. 2012; Thornicroft *et al*. 2012; Ng *et al*. 2016; Beran *et al*. 2017). The aim of this paper is to describe these modifications, the content of our course for a group of LMIC early-career researchers and early feasibility outcomes.

### Development

The course's starting point was a career development series for early-career faculty called THRIVE at the Institute of Psychiatry, Psychology and Neuroscience, King's College London (KCL), UK (King's College London, 2017). THRIVE was based on professional development workshops from medical schools at several US universities, which were designed to build non-technical research competencies (Iversen, personal communication).

First, AMARI KCL staff (MA, CM, HJ) met KCL academics who had developed THRIVE to discuss the course content, which comprised eight face-to-face sessions. Next, they consulted AMARI project staff in Ethiopia, Malawi, Zimbabwe and South Africa on local needs and resources. These consultations highlighted the need for modifications to THRIVE, both logistically (e.g. delivering some sessions via web-based platforms) and culturally (for instance, the work–life balance and career strategy sessions needed to be modified to be appropriate for local needs by ensuring that personal examples were taken from African researchers to be relevant to AMARI fellows). Specific adaptation details are provided for individual sessions below.

Further planning meetings were arranged by KCL project staff with external UK-based consultancy firms to provide expert input on training for mentoring (Get the Picture Consulting) and presentation skills (Westbourne Consulting). This included ‘train-the-trainer’ sessions for the KCL programme leader and negotiation over rights to use consultants' intellectual property (training materials and manuals) with AMARI fellows during the project and beyond.

The course was named the Academic Competencies Series (ACES). A draft plan for the course was presented to the AMARI Steering Committee in November 2016 and approved following a final discussion of needs, which recognised both the requirement for local contextualisation and the benefit of drawing on external experience from those involved with THRIVE. This dialogue between KCL and AMARI colleagues in the SSA countries led to two additional workshops being introduced to meet local needs: (1) academic writing for publication, and (2) engaging policy-makers. Delivery of the first ACES course began in March 2017 in Zimbabwe and continued until March 2018, running through 10 separate sessions (Table 1). ACES will then repeat from 2018 to 2019 for the next cohort of AMARI research fellows.

**Table 1.** ACES workshops and facilitation details

No.	Workshop title	Thematic area	Delivery method	Facilitation method	Facilitator's background
1	Mentoring	Self-development	Face to face	Train the trainer/HIC	HIC programme manager with LMIC experience
2	Teamwork	Self-development	Face to face	HIC/LMIC co-led	HIC programme manager/LMIC senior researchers and clinicians
3	Work–life balance	Self-development	Face to face	HIC/LMIC co-led	HIC programme manager/LMIC senior researchers and clinicians
4	Career planning	Self-development	Face to face	External consultant/HIC	HIC and LMIC experience; briefed for specific context
5	Presentation skills	Engagement	Face to face	Train the trainer/HIC	HIC programme manager with LMIC experience
6	Digital media	Engagement	Webinar	External consultant	No LMIC-specific experience but briefed for context
7	Policy makers	Engagement	Webinar	External consultant	No LMIC-specific experience but briefed for context
8	Teaching	Engagement	Webinar	HIC-led with LMIC experience	HIC clinical academic with LMIC experience
9	Grant applications	Writing skills	Webinar	HIC/LMIC co-led	Senior academics from HIC and LMIC
10	Academic papers	Writing skills	Face to face	HIC-led with LMIC experience	HIC clinical academic with LMIC experience

### Implementation

The original THRIVE course was delivered in half-day monthly workshops in London. However, because AMARI covers four African countries and monthly gatherings are neither practical nor cost-effective, a more flexible approach was required for ACES. AMARI KCL staff consulted the organisers of THRIVE on which sessions were best for in-person delivery, and which could be suitable for web-based delivery. We then modified delivery to combine face-to-face and web-based sessions, many of which were co-taught with African partners (Table 1 describes the delivery method for each session). Six sessions were delivered face-to-face, and five of these were scheduled alongside other AMARI events to minimise travel costs (flights, visas, etc.). The other session (academic writing) was a standalone residential workshop. Overall the course was delivered over a period of 1 year.

The online platform chosen for webinar delivery was WebEx (Cisco Systems, San Jose, CA). WebEx was tested for functionality across all AMARI countries before purchase and a user guide produced for fellows. Upgraded storage capacity was obtained to allow all sessions to be recorded and streamed. This enabled anyone who could not attend live to stream a recording of the webinar later.

AMARI research fellows were surveyed anonymously online via SurveyMonkey to ascertain their availability for webinar sessions. A regular time slot was selected in accordance with the clear majority choice, which allowed webinar sessions to be scheduled months in advance. Webinars were facilitated live by the ACES programme leader who set up the session, introduced the speaker, timed for breaks and remained on email throughout the webinar in parallel to troubleshoot access and connectivity issues for fellows. All webinars were interactive with question and answer sessions facilitated by the programme leader, using direct speech or typing text questions using WebEx's ‘Chat’ feature. All course sessions were compulsory and attendance was monitored by the programme leader.

### Content

This section describes all 10 components of the course, with a general protocol for delivery, broken down by three thematic areas: (A) self-development, (B) engagement and (C) writing skills.

- *Self-development*: These sessions focused on self-management and personal development, including through mentoring, and were aimed at helping researchers to manage stress, time, professional relationships and career plans over the longer term.
  1. *Mentoring*: This day-long face-to-face session introduced the theory of developmental mentoring, contrasting it with traditional (classical) mentoring, and examining research on evidence for the effectiveness of mentoring. Training covered both mentor and mentee roles, and fellows were given manuals for their roles as a mentor or mentee. This training was geared towards preparing trainees to participate in a peer mentoring programme, which consisted of six formal sessions of 1 h, spaced over 1 year, where the second year AMARI fellows mentored first year fellows, aiming to draw on the benefit of their experience in the programme to date. Pair and small group exercises gave opportunities for experiential learning through practising mentoring conversations, particularly using the ‘GROW’ model (Goal, Reality, Options, Will/Wrap up/Way forward) (Whitmore, 2017). The peer mentoring programme is still running at the time of writing.
  2. *Teamwork*: This three-quarter day face-to-face session introduced concepts and theories around working in a team, with examples drawn from scientific environments such as research laboratories and university departments. Fellows engaged in two extended role-play scenarios which were specially written by the ACES programme leader for this workshop, drawing generally on experiences of AMARI fellows and developed in consultation with AMARI country leads. The role-plays addressed two common difficult situations in team environments: (1) securing commitment from a busy senior supervisor to review your work before a deadline, and (2) confronting a junior colleague on plagiarism (full text of scenarios available in online Supplementary Appendix 2). There was a facilitated feedback session after the role-plays to discuss experiences and learning. After this, each fellow took a Myers–Briggs Type Indicator (MBTI) Step I assessment (Briggs & Myers, 1987) led by an accredited assessor who was also a member of the AMARI Zimbabwe team, and these were scored in-session. Discussion was then facilitated on how ‘types’ fit into teams, individuals' adaptation to change in teams and workplace conflict management.
  3. *Work–life balance*: This three-quarter day face-to-face session covered theory on time management, offering practical tools for organisation (memory aids, Gantt charts) and prioritisation (Eisenhower matrices). This was followed by extended question-and-answer sessions with two senior African academics (one male, one female) who discussed how they handled work–life balance challenges, such as extended family demands in the SSA context (where people are often expected to help cousins and other relatives by giving time, advice and financial support), community status, dilemmas of moving to high-income settings to work, developing international networks and the challenges of building a career in newer research institutions. These rich discussions were supplemented with brief video interviews recorded prior to the session with senior academics from both HIC and LMIC settings who provided additional tips on finding balance in a busy research career.
  4. *Career planning*: This day-long face-to-face workshop was designed to help fellows make the transition from novice researcher to research leader. The day began with a discussion around what makes the world of research challenging today, as well as defining ‘success’ in research. Key principles of managing a research career were then introduced: setting goals and priorities (both professional and personal), making a plan and activating mentoring and other relationships to assist in these objectives and positioning oneself for overall research career success. The workshop included self-reflection and both small and large group discussion. It aimed to build a sense of personal agency among the AMARI fellows for their own career planning and management. This workshop was run by a world expert in academic and research career management.
- *Engagement*: These sessions were themed around how to communicate research findings to both specialist and non-specialist stakeholders to maximise impact and implementation.
  1. *Presentation skills*: This day-long workshop centred on giving effective oral presentations with visual support (e.g. PowerPoint slides). After an introduction to the topic, two fellows volunteered to give short presentations. The facilitator led a group discussion on aspects of delivery (voice, body language, pace, audience engagement), based on peer feedback to the two presenters. Further sections covered structuring a talk using ‘storytelling’ techniques, and producing clear, effective and visually-engaging slides to accompany a talk. Each fellow had to give a presentation at a scientific conference the week of this training. They had prepared presentations in advance of the workshop and each received 20 min personal input from the facilitator. These individual consultations were given by the ACES programme leader (trained by Westbourne Consulting, UK), and included feedback on oral presentations and personal guidance tuition on visual slides.
  2. *Digital media*: This half-day webinar focused on assisting fellows to disseminate their own research findings using digital media, and the use of digital media to engage in research discourse. The main topics covered were tweeting, blogging, online security and contact with the media. Digital publishing and open data platforms were also discussed. This webinar was led by a UK-based neuroscientist who is an expert in popular science writing and blogging and has written tens of features for national and international press as well as hosting a blog and Twitter page with nearly 50 000 followers.
  3. *Engaging policy makers*: This half-day webinar covered key themes in public mental health and the challenge of reducing treatment and research gaps. It highlighted how policy makers gather information and make decisions about public health and how best to influence their thinking using research evidence, such as by presenting data on economic outcomes of public health interventions. The webinar drew from case studies from the UK and a new case study designed in partnership with AMARI African partner sites. The workshop was led by a UK-based psychiatrist with several years' experience in policymaking for the UK government.
  4. *Teaching*: This half-day webinar covered the skills needed to deliver effective teaching. The theory of teaching adults, small *v.* large group teaching, as well as methods for evaluating teaching, were addressed in brief didactic sessions interspersed with discussion. Adapting teaching for LMIC settings was also described, including running webinars via chatrooms where bandwidth does not allow audio or video participation. The facilitator of this webinar was a UK psychiatrist based in SSA, with extensive medical education experience in Sierra Leone and Somaliland. The session was highly interactive and drew on the broad existing teaching experience among the cohort of AMARI fellows, many of whom were involved in delivering courses to undergraduate and master's students.
- *Writing skills*: These workshops were aimed at developing writing skills in two formal situations to enhance fellows' abilities to obtain research funding and to disseminate their work in peer-reviewed publications.
  1. *Grant applications*: This half-day webinar covered the basics of grant writing, focusing on how to think like a reviewer to increase the chances of a favourable review. A didactic session covered the key sections of an application with tips on developing and presenting proposals. This was followed by a discussion between fellows and facilitators (both HIC and LMIC) on successes and failures in grant writing.
  2. *Academic papers*: This 5-day experiential workshop combined protected time for writing with interactive didactic sessions on structuring academic papers, the logistics of publishing and grammar and style. Each fellow brought a manuscript to develop and engaged in peer-editing with other fellows to exchange comments on drafts. The workshops were designed and facilitated by a US-based clinical academic with extensive publication experience. The full schedule is shown in online Supplementary Appendix 3.

## Results

### Delivery

Attendance was high, with a mean average of 90%, and is described in detail in Table 2. Four of the 10 workshops were delivered in a webinar format, while the six face-to-face workshops were delivered across four locations in three SSA countries.

**Table 2.** Delivery statistics for the first round of ACES training

Workshop title	Delivery date	Location	Session length	Number of attendees^a^/eligible fellows	Attendance % of eligible
Mentoring	March 2017	Victoria Falls, Zimbabwe	1.0 day	18/18	100
Presentation skills	March 2017	Victoria Falls, Zimbabwe	1 day	18/18	100
Digital media	May 2017	Web-based	0.5 day	16/18	88.9
Teamwork	June 2017	Hawassa, Ethiopia	0.75 day	14/16	87.5
Work–life balance	June 2017	Hawassa, Ethiopia	0.75 day	14/16	87.5
Teaching	September 2017	Web-based	0.5 day	14/16	87.5
Policy makers	October 2017	Web-based	0.5 day	13/16	81.3
Grant applications	November 2017	Web-based	0.5 day	15/16	93.8
Academic papers	November 2017	Blantyre, Malawi	5 days	12/16	75.0
Career strategy	March 2018	Lilongwe, Malawi	1 day	16/16	100
				Mean average:	90.2

a During the first three workshops, there were 18 fellows on the programme. However, in late May 2017 two fellows dropped out of AMARI (one for personal reasons, the other due to programme eligibility), making the subsequent number of eligible fellows 16.

### Evaluation

Full evaluation of the course will be undertaken separately. This will include quantitative data (self-rated skills before and after each session), collected as part of an AMARI-wide monitoring and evaluation scheme, as well as qualitative data, obtained through semi-structured interviews and analysed using Thematic Analysis. The mentoring scheme will be evaluated separately using anonymous online surveys conducted at baseline, 6 months and 1 year of the mentoring scheme. This evaluation is scheduled for early 2020 following completion of all AMARI training activities in late 2019.

### Cost

Table 3 provides a broad summary of the costs associated with running this course for 1 year, i.e. through one complete cycle, delivering each workshop once to all 16 AMARI fellows enrolled at the time of conducting the course. The total cost was approximately US$72 520. This is equivalent to US$4530 per fellow. The most expensive component was staff costs, accounting for nearly US$39 000; this included 1.5 days/week salary for the ACES programme manager for 1 year, as well as consultancy payments. However, this also comprised one-off costs such as designing and setting up the course, meaning that in subsequent years, costs would be lower for delivering a second round of workshops.

**Table 3.** Costs for delivering ACES course over 1 year for 16 fellows

Cost	Amount (GBP/£)	Amount (USD/$)
Staff costs including post-doc programme manager (0.3 FTE) and consultants	29 800	38 740
Staff travel, accommodation, visas and subsistence (face-to-face workshops)	15 300	19 890
Fellows’ travel, accommodation, visas and subsistence (face-to-face workshops)	8384	10 900
Intellectual property fees (licensing of workshop materials)	1000	1300
Test materials (Myers–Briggs test forms and question booklets) and other printing	700	910
WebEx subscription with 10GB additional storage capacity for streaming video	600	780
Total	55 784	72 520

All costs to nearest £100 or $100; USD calculated at an approximate rate of 1.3 USD  =  1.0 GBP, correct at time of writing (September 2018).

FTE  =  Full Time Equivalent (i.e. 0.3 FTE  =  1.5 days per week).

### Opportunities

In addition to high attendance and relatively low cost, workshops offered valuable opportunities for networking between fellows, including internationally between different project countries. Fellows were typically engaged in the skills they were learning outside of the sessions. Many were actively presenting, mentoring, disseminating research, working in teams and teaching. This offered them the chance to implement their learning quickly.

### Challenges

There were also some challenges experienced in running the workshops series:

1. *Adaptation*: The original HIC course, THRIVE, on which this course was based, was not formally adapted to the LMIC/SSA context following an adaptation protocol or methodology. Ideally, the course would have benefitted from a full needs assessment, greater consultation and small-scale piloting of materials before delivery to a cohort of research fellows. Formal adaptation would have ensured that all course materials were maximally relevant to local needs. However, this was not practicable given time constraints within project and recruitment deadlines.
2. *Attendance*: Though generally high at 90%, attendance was not 100% because fellows had professional and personal commitments which could not be avoided. This reflects the reality of the context where people have multiple roles (clinician, NGO worker, government employee, parent, extended family member) alongside their status as a researcher. It was considered important to let fellows manage these demands due to, for example, the pressing need for clinical services which many fellows provide in their countries. These multiple demands and roles also affected some fellows' ability to devote five full days (plus travel time) for the academic writing workshop, which had the lowest attendance at 75%. Those unable to attend the webinars were encouraged to watch recordings of sessions. There is no way of monitoring whether this has occurred, but since the fellows are adult learners, they hold responsibility for setting own priorities and can, to some extent, tailor their involvement accordingly, e.g. watching a recording on grant writing later in the course of their Ph.D., when they come to write a funding application.
3. *Technical issues*: Some fellows experienced problems with connectivity during the webinars. Mostly these related to occasional low Wi-Fi strength or low Ethernet bandwidth, particularly where fellows attended from non-institutional locations, e.g. home. Fellows unable to connect reliably were advised to find an alternative location next time with better connectivity, or ‘buddy up’ with another fellow who had a proven high-quality connection. In later webinars, fellows often co-located to take advantage of reliable Ethernet and Wi-Fi.

## Discussion

As part of an international research capacity building project in SSA, we implemented a course for early career researchers to develop a range of broader academic competencies to complement their scientific and technical knowledge and help position themselves for research success. To our knowledge, it is the first training package to be delivered in SSA that combines all of these components in one course.

The ACES course also used a novel combination of HIC and LMIC facilitation, which enabled modifications to be made for the African context, both in terms of content and delivery, in line with literature on best practice in research capacity building (Whitworth *et al*. 2008; Thornicroft *et al*. 2012). The logistical constraints of face-to-face training for participants and facilitators from five SSA countries (four AMARI countries plus Kenya) and three other partner countries (UK, USA, Australia) necessitated flexible delivery methods. Use of a video conferencing platform enabled a greater number and range of workshops to be delivered. The webinar format also allowed research fellows with busy professional roles (e.g. as clinicians) to work flexibly around their schedules, streaming recorded sessions at a convenient time. The face-to-face sessions in SSA allowed non-AMARI local collaborators to attend at host sites, offering further networking possibilities.

In addition to the challenges described above, there are further limitations to this report of the capacity-building course. Firstly, the course has not yet been formally evaluated. Though the delivery, attendance and cost data provided here indicate that implementing the course is feasible, no formal data exist yet on acceptability. Impact on career success would be a useful outcome, despite the difficulty of measuring it reliably (Cochrane *et al*. 2014). Evaluation plans were described here for qualitative and quantitative assessment; these will be reported separately when complete. Secondly, there is a need for sustainability and legacy of training knowledge in project countries. Plans are being drawn up for ‘train the trainer’ sessions to be run towards the end of the project. Expert consultants who led workshops on this course will train researchers from SSA partner countries, who in turn can disseminate training sustainably within their own institutions and research networks, beyond the lifespan of the immediate project. Despite these limitations, we believe that the protocol, delivery and cost data provided here indicate that such a career-development research capacity building course is feasible for implementation in SSA.

For researchers to maximise their career development opportunities, they need to be operating within strong systems at the institutional and sub-national/national levels (Thornicroft *et al*. 2012; World Health Organisation, 2016; Semrau *et al*. 2018). To that end, the AMARI consortium is also undertaking a large-scale assessment of health and research systems in the four SSA partner countries, with the aim of identifying barriers and enablers for career progression and sustainability for young mental health researchers in these LMICs. This will be used to advocate locally for change. The ACES course described here can be one way of contributing to that larger, over-arching aim of capacity building to reduce the research and treatment gaps in LMICs, particularly in SSA.

## Conclusions

This study described the contextualisation and implementation of an innovative capacity building course for career development skills to support early-career researchers from four SSA countries. Course content focused on self-development, engagement and writing. The course was well-attended, delivered at relatively low cost and offered additional opportunities such as networking for participants. Though it is yet to be formally evaluated, these logistical outcomes suggest it would be feasible for delivery in other LMIC settings as part of research capacity building programmes.

## Supplementary material

For supplementary material accompanying this paper visit http://dx.doi.org/10.1017/gmh.2019.14. click here to view supplementary material
