# Supplementary material for: Positioning for success: building capacity in academic competencies for early-career researchers in sub-Saharan Africa
Source: Glob Ment Health (Camb). 2019 Jul 19;6:e16. doi: 10.1017/gmh.2019.14 (PMC6669964; doi:10.1017/gmh.2019.14)
Supplement: Supplementary file 1 [file gmhsup.zip › S2054425119000141sup003.docx]

**Appendix 3: Academic writing workshop schedule**

**AMARI – Manuscript Writing Workshop**

**Timetable**

***Course objectives:***

- Participants will make substantial progress on their manuscript and will develop a concrete, manageable plan and timeline for getting it ready for submission
- Participants will understand the publication process and the elements of a successful academic manuscript
- Participants will understand other modalities for disseminating data and the limitations of academic publication

**Day 1:** Monday, November 20 (5 PM to 6 PM)

*Prior to the start of the workshop, please read Chapter 3 “Tell a story” in Writing Science in Plain English and try the first two of each exercise.*

| 5:00 – 5:30 | Introductions  Workshop overview and approach |
| --- | --- |
| 5:30 – 6:00 | Review: Talking about writing (Refer to Appendix 1 in *Writing Science in Plain English*) |
| 6:00 – 6:15 | Introduce writing book and exercise to complete for tomorrow |
| 5:30 – 7:30 | One-on-one meetings  *During the first two days of the workshop, all participants will have one 30-minute meeting with Helen to discuss:*   - *What paper you are focusing on* - *Current status of that paper* - *What you hope to have done on that paper by the end of the week* - *Day by day work plan* - *Communication with supervisor* |

For Tuesday, please read the chapter “Tell a story” in *Writing Science in Plain English* and try the first two of each exercise

**Day 2:** Tuesday, November 21 (9 AM to 6 PM)

| 9:00 – 10:00 | Exercise: Making each paragraph have a job |
| --- | --- |
| 10:00– 11:00 | Writing skill: “Place subjects and verbs close together”: Review, practice exercise, and application  Writing skill: “Make characters subjects and their actions verbs”: Review, practice exercise, and application  From *Writing Science in Plain English* |
| 11:00 – 12:30 | Work time  One-on-one meetings |
| 12:30-1:30 | Lunch |
| 1:30 – 2:00 | Structure skill: Writing an introduction |
| 2:00 – 3:30 | Writing an introduction group exercise |
| 3:30 – 4:30 | Work time  One-on-one meetings |
| 4:30 – 5:30 | Structure skills: Using prior research – synthesis, not summary |
| 5:30 – 6:00 | Writing skill: “Use strong verbs”: Review, practice exercise, and application  From *Writing Science in Plain English* |

For Wednesday, please read the chapter “Favor the active voice” in *Writing Science in Plain English*

**Day 3:** Wednesday, 22 November (9 AM to 6 PM)

| 9:00 – 9:45 | Publication skill: Working with supervisors and authorship teams |
| --- | --- |
| 9:45 – 10:00 | Writing an abstract |
| 10:15 - 12:30 | Write your abstract – send it to Helen when you are done  Then work time and one-on-one meetings |
| 12:30 – 1:30 | Lunch |
| 1:30 – 2:00 | Structure skill: Writing a discussion section |
| 2:00 – 4:00 | Writing a discussion – group exercise |
| 4:00 – 5:00 | Work time  One-on-one meetings |
| 5:00 – 6:00 | Writing skill: “Favor the active voice”: Review, practice exercises, and application  Writing skill: “Make lists parallel”  From *Writing Science in Plain English* |

For Thursday, please read the chapter “Arranging your paragraphs” in *Writing Science in Plain English*

**Day 4:** Thursday, 23 November (9 AM to 6 PM)

| 9:00 – 9:30 | Publication skill: Writing a cover letter |
| --- | --- |
| 9:30 – 11:45 | Write your cover letter and send to Helen  Then work time and one-on-one meetings |
| 11:45 – 12:30 | Structure skill: “Arranging your paragraphs”: Review and application |
| 12:30 – 1:30 | Lunch |
| 1:30 – 3:00 | Work time  One-on-one meetings |
| 3:00 – 4:00 | Writing skill: “Omit needless words” |
| 4:00 – 5:30 | Structured peer editing |

For Friday, please read the chapter “Old information and new information” in *Writing Science in Plain English*

**Day 5:** Friday, 24 November (9 AM to 6 PM)

| 9:00 – 9:45 | Publication skill: What are you going to do besides publish? |
| --- | --- |
| 9:45 – 11:15 | Work time  One-on-one meetings |
| 11:15 – 12:30 | Structure skill: Writing the methods section |
| 12:30 – 1:30 | Lunch |
| 1:30 – 2:30 | Writing skill: “Old information and new information”: Review, practice exercises, and application |
| 2:30 – 4:00 | Work time  One-on-one meetings |
| 4:00 – 5:00 | Writing skills review and practice |
| 5:00 – 6:00 | To be determined based on needs that arise during the course |

**Day 6:** Saturday, 25 November (9 AM to 3 PM)

| 9:00 – 10:00 | Structure skill: Results section |
| --- | --- |
| 10:00 – 12:00 | Work time  One-on-one meetings |
| 12:00 – 12:30 | Writing skills: To be determined based on needs that arise during the course |
| 12:30 – 1:30 | Lunch |
| 1:30 – 2:00 | Publication skill: Formatting and submitting your paper |
| 2:00 – 3:00 | Development of personalized plans for taking paper to publication and drafting email to supervisor about personalized plan |
