# Supplementary material for: Positioning for success: building capacity in academic competencies for early-career researchers in sub-Saharan Africa
Source: Glob Ment Health (Camb). 2019 Jul 19;6:e16. doi: 10.1017/gmh.2019.14 (PMC6669964; doi:10.1017/gmh.2019.14)
Supplement: Supplementary file 1 [file gmhsup.zip › S2054425119000141sup002.docx]

**Appendix 2: Teamwork Role Plays**

**Role play scenario 1**

In pairs – one fellow takes each role. Take a few minutes to read the instructions on your role. Run the conversation for 10 minutes. Then swap over which of you plays the AMARI fellow, and try the other scenario.

**AMARI fellow (PhD/Post-Doc) brief:**

- Your PhD supervisor has been difficult to get hold of recently. They always seem to be busy and you feel that they are not devoting enough time to your work. Recently you sent them a draft of your literature review, but when you asked them about it two weeks later they had not read it. You thought you had a meeting to discuss it with them last week, but when you emailed to confirm, you got an Out of Office message saying they were overseas at a conference. You know they have been working hard on a grant application but you need their input to the literature review draft in order to make progress in your PhD. Today you have managed to get ten minutes with them before they have to give a lecture in the department. Your aim is to explain your concerns to them diplomatically, and get them to agree to some kind of change in the way you communicate, to make working with them more efficient and less frustrating!

**PhD Supervisor brief**

- You are a very busy academic in the department. You have many research projects on the go, you are supervising five postgraduate students and you have a large grant application in progress, on top of a full teaching schedule. And you have two children at home – one of them has been ill recently and this has put you under more strain. There are not enough hours in the day! Before you are due to give a lecture, one of your PhD students has asked to speak to you. You think that there was something you needed to do for them, but can’t remember exactly what – they didn’t explain it very clearly. You didn’t know what their deadline was for the work. You know that you need to make more time to deal with them, but right now the main thing on your mind is the lecture you need to give in ten minutes’ time. Your aim is to let the PhD student know how busy you are, and that if they want something done they need to be clear about exactly what they want from you, and when they want it done. Listen to their concerns and see if you can reach an agreement with them about future communication.

**Role play scenario 2**

In pairs – one fellow takes each role. Take a few minutes to read the instructions on your role. Run the conversation for 10 minutes. Then swap over which of you plays the AMARI fellow, and try the other scenario.

**AMARI fellow (PhD/Post-Doc) brief:**

- You asked a Research Assistant who has been working with you to produce a report for you to summarise new developments in your academic field. However, when they presented their report to you, something about it looked familiar… You checked the text on the internet and it turned out that they had copied large sections of their report from someone else’s work on the internet. You have asked to meet them to discuss this. Explain what you have discovered about their report. Try to find out why it happened. Explain to them that this is plagiarism and it is not ok. Try to agree with them that they will not do this in the future and ensure that they understand why it is not acceptable in science (even in ‘informal’ reports). Try to find ways to motivate them (e.g. by giving them praise for other work they’ve done) to leave them feeling positive at the end of the meeting.

**Research Assistant brief**

- You are a Research Assistant in the department. Your supervisor has asked to speak to you about a report which you wrote for them last week. You don’t know why, but it didn’t sound like they were happy. Maybe you wrote the wrong thing, or the information in your report was incorrect? You had lots of other demands on your time that week, so you used the Internet to help you with the report. You copied sections from other similar documents in order to save time – if someone else has already done the work then why should you need to repeat it? Also, it was only a small briefing for your boss so it didn’t really matter. You just want to be sure you’re not in trouble. Try to defend yourself if your boss is giving you a hard time! After all, you haven’t done anything wrong…
