# Supplementary material for: Positioning for success: building capacity in academic competencies for early-career researchers in sub-Saharan Africa
Source: Glob Ment Health (Camb). 2019 Jul 19;6:e16. doi: 10.1017/gmh.2019.14 (PMC6669964; doi:10.1017/gmh.2019.14)
Supplement: Supplementary file 1 [file gmhsup.zip › S2054425119000141sup001.docx]

**Appendix 1: AMARI Fellows’ Recruitment Process**

- PhD and Post-doc fellowships were advertised using a variety of methods, including internally at faculties, online, and in national newspapers, for a minimum of 4 weeks in each AMARI country. Applications received were rated by a committee consisting of local academics and clinicians, as well as an external AMARI representative, according to relevance of topic, publications, motivational statement, proposal evaluation and gender. Specific shortlisting criteria were as follows:
  - Demonstrated interest in research
  - References of conducted research
  - Academic language
  - Academic qualifications
  - Potential/capacity for leadership
  - Quality of proposal submitted (including absence of plagiarism)
  - Gender (additional weighting for female candidates)
- Shortlisted candidates for each institution were then interviewed by a combination of local institution faculty and external academics (drawn from the wider AMARI team or from senior academics within the university). The top-rated candidates were offered an AMARI fellowship (numbers at each university varied by country depending on funding and targets). This process was repeated each year until recruitment targets were met.
- MPhil fellowships in Public Mental Health were advertised through all institutions using same methods as PhD/Post-doc adverts. The MPhil fellows were registered at the University of Cape Town in South Africa but were open to candidates from any AMARI country. Five places were available per year for four years, with a total target of 20 MPhil fellowships. One MPhil place per year was reserved for a Malawian candidate, and one per year for a Zimbabwean candidate, to ensure equity of access to the MPhil fellowship across project countries. The remaining three fellowships per year were open to applicants from all AMARI countries. All MPhil applications were sent to two independent reviewers and presented to a local selection committee. The local selection team finalised the shortlist before conducting interviews in person or via online video call (depending on candidate location). The interview panel consisted of senior management of the Alan J. Flisher Centre for Public Mental Health. Successful candidates were then offered the MPhil fellowship.
